# Balance Maintenance in High-Speed Motion of Humanoid Robot Arm-Based on the 6D Constraints of Momentum Change Rate

**DOI:** 10.1155/2014/535294

**Published:** 2014-04-17

**Authors:** Da-song Zhang, Rong Xiong, Jun Wu, Jian Chu

**Affiliations:** National Laboratory of Industrial Control Technology, Zhejiang University, Hangzhou, Zhejiang 310027, China

## Abstract

Based on the 6D constraints of momentum change rate (CMCR), this paper puts forward a real-time and full balance maintenance method for the humanoid robot during high-speed movement of its 7-DOF arm. First, the total momentum formula for the robot's two arms is given and the momentum change rate is defined by the time derivative of the total momentum. The author also illustrates the idea of full balance maintenance and analyzes the physical meaning of 6D CMCR and its fundamental relation to full balance maintenance. Moreover, discretization and optimization solution of CMCR has been provided with the motion constraint of the auxiliary arm's joint, and the solving algorithm is optimized. The simulation results have shown the validity and generality of the proposed method on the full balance maintenance in the 6 DOFs of the robot body under 6D CMCR. This method ensures 6D dynamics balance performance and increases abundant ZMP stability margin. The resulting motion of the auxiliary arm has large abundance in joint space, and the angular velocity and the angular acceleration of these joints lie within the predefined limits. The proposed algorithm also has good real-time performance.

## 1. Introduction

Humanoid robot is characterized as a high-order, nonlinear, and multiple DOF unstable system. Research topics such as motion planning and balance maintenance for humanoids remain a great challenge. After years of development, there have already been several humanoid robot systems with outstanding performance, such as WABIAN of Waseda University [1], ASIMO of Honda Inc. [2], QRIO of Sony Inc. [3], HRP of AIST [4], and HUBO of South Korea [5]. With the advancing progress in both theoretic study and engineering practice, humanoid robot is evolving towards a higher level of intelligence and mobility. Nowadays humanoids can display more complex motions, such as pushing a table [6, 7], fetching and manipulating small objects [8], assembling under industrial environment [9], lifting a heavy object with human help [10], and dancing featuring with whole-body movement [11]. These various complicated dynamic operations bring the robot body time-varying impact in both force and moment, which makes the balance maintenance a critical and important research issue for the humanoid robots.

Balance maintenance is the key to successful humanoid robot, which is fundamentally unstable. Instinct makes us believe that balance comes along with low velocity; in other words, low momentum can guarantee balance. However, we also notice that high-speed trains, airplanes, and rockets can operate steadily while their momentum is large. Why do we have the illusion that balance comes along with lower momentum? As a matter of fact, objects with lower momentum usually need a lower momentum change rate when they switch from a moving state into stationary within a unit period of time or vice versa. Put in other words, the momentum change rate is smaller. In fact, the momentum change rate of high-speed trains, airplanes, and rockets in their moving process is either quite low or is rigorously controlled within certain safe limits, even though their momentum is extremely large. For instance, the high-speed trains will cover a long distance to accelerate or decelerate when it is starting or braking, which actually is to decrease the acceleration or the deceleration; in other words, the momentum change rate of the train in the whole process of its drive is controlled within a small range. Moreover, planes and rockets are often equipped with accelerometers, which are used to monitor their acceleration and deceleration and thus help control them within a certain safe range. Serious accidents caused by full braking are in fact resulted by the overly great deceleration; in other words, the momentum change rate is too big. Therefore, the key to perform balanced and steady motion lies in acceleration control or the momentum change rate control more strictly. For humanoid robots, the essence of balance maintenance also depends on the regulation of the momentum change rate. Equally speaking, the key is the control of the ground reaction force and moment to the robot.

Based on the constraints of momentum change rate, this paper sets out to study the real-time balance maintenance utilizing the auxiliary arm of the robot during high-speed arm motions. Different from the industrial robot, which is located fixed on the ground, the humanoid robot is “pressed” to the ground by gravity. Thus, the 6D time-varying inertia impact generated by the high-speed motion of the robot arm might cause the robot tilt, slide, or even jump, which leads to the shifting of the end point of the operating arm and failure of the manipulator task. The force and moment which the robot exerts to the ground should be controlled within a safe range by properly designed motion of the auxiliary arm and the resultant force and moment impact due to the fact that high-speed motion of the operating arm should be eliminated or reduced, so as to guarantee the accurate and stable operation of the humanoid robot.

A balance maintenance method based on resolved momentum control has been adopted in prior studies [12, 13]; however, the high-speed motion of the robot arm only makes the method applicable to the balance maintenance on no more than two dimensions and inapplicable on three or more dimensions. According to Newton's second law and momentum conservation principle, the authors put forward an optimal balance maintenance method that generates proper motions of the auxiliary arm's joint based on the constraints of momentum change rate (CMCR). From the perspective of CMCR, the authors first give the formula to calculate the total momentum of the motion of the robot's two arms. Then the fundamental relation between 6D momentum change rate and full balance is specifically examined. In addition, the discretization process of full balance maintenance under the constraint of 6D momentum change rate is provided along with the convex quadratic programming equation constrained by inequalities. The angular velocity and angular acceleration constraints on the joints of the auxiliary arm are considered at the same time. The angular velocity vector of optimal joint motion of the auxiliary arm under CMCR and the constraints of auxiliary arm's motion are also introduced. Lastly, the authors explain the methods taken to increase the algorithm speed. The simulation experiments show that the algorithm is effective and has good real-time performance.

## 2. Related Works

The balance maintenance method in the early years was centered on static balance maintenance, which keeps the projection of center of gravity within the support area. In the year of 1972, the Yugoslav scholar Dr. Vukobratovic put forward the ZMP (Zero-Moment Point) theory [14], which has become the basic theoretic foundation of the dynamic balance maintenance of humanoid robots. At present, ZMP control method has been widely applied in the dynamic walking balance maintenance of humanoid robot, for example, the most famous ASIMO. ASIMO, equipped with the world's most advanced technologies, features a systematic ZMP control scheme, including ground reactive force control, model ZMP control, and foot landing position control [2].

In recent years, researchers from different fields have undertaken deep studies on motion planning and balance maintenance from various perspectives as humanoid robot become more and more popular. In particular, balance maintenance of humanoid robot based on momentum control has caught great attention. As for linear momentum control, balance maintenance generally requires that the projection of the center of gravity falls within the support area by controlling the linear momentum [15, 16]. From the perspective of angular momentum control, it has been learned that the central nervous system of human beings would intentionally adjust the angular momentum with respect to the center of gravity while walking, and thus the method of minimizing the angular momentum is proposed [17, 18]. Besides, the stability of the robot is also evaluated using the criterion that the rate of angular momentum change should be zero [19].

To achieve better balance maintenance performance, Professor Shuuji Kajita at AIST (National Institute of Advanced Industrial Science and Technology) of Japan put forward in 2003 the resolved momentum control scheme considering both the linear momentum and angular momentum based on his long-time research results and technological accumulations [13]. He applied this control strategy to achieve whole-body autonomous balance maintenance of the humanoid robot HRP-2 under remote operation. Several years later, he succeeded in autonomous balance of the robot when it picked objects up from the ground [12].

However, it has been found in practice that (1) when the resolved momentum control is applied to high-speed 6D full balance maintenance, the angular velocity of the auxiliary arm may exceed its limit; and the overrun is so great that it further leads to the overrun of angular acceleration of the joints immediately; (2) when the resolved momentum control is applied to high-speed and low-dimensional balance maintenance problem, the effect of the balance maintenance is excellent in the specified dimensions, but has no positive result on the other dimensions. For instance, when ZMP is regarded as the performance index of the balance maintenance problem, in other words the forward and backward tilt and left and right tilt dimensions are considered, the best ZMP balance can be achieved using the resolved momentum control and the tilting of the robot in these dimensions is well controlled. Nevertheless, the balance of other dimensions not considered in the scheme is totally lost, which leads the robot to slip and jump. The close relation between humanoid robot's balance and its momentum change rate is clearly illustrated by Newton's second law. The authors propose an optimal, real-time, all-dimensional balance maintenance method, based on CMCR, to meet the needs of the 7-DOF arm of the robot to perform high-speed dynamic operation safely.

## 3. Balance Maintenance of the Auxiliary Arm

### 3.1. Total Momentum Calculation

**Figure 1:**
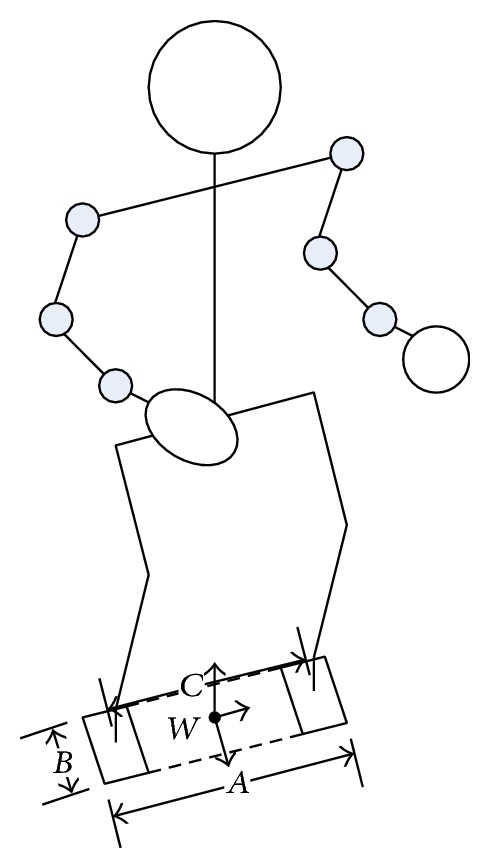
Humanoid robot model.

This paper assumes that the humanoid robot moves only its two arms to perform certain tasks while the other parts of the body are fixed with no movement. Therefore, the total momentum of the robot equals the total momentum of its two arms. As shown in Figure 1, the origin of the reference coordinate is selected as the central point *w* of the two feet. By referring to the resolved momentum control, the total momentum, including linear momentum *P* and angular momentum *L*, can be defined as [13]

(1) $$\begin{array}{c} \left[\begin{array}{c} P\\ L \end{array}\right]=\left[\begin{array}{c} M_{\text{jobarm}}\\ H_{\text{jobarm}} \end{array}\right]{\dot{\theta }}_{\text{jobarm}}+\left[\begin{array}{c} M_{\text{aidarm}}\\ H_{\text{aidarm}} \end{array}\right]{\dot{\theta }}_{\text{aidarm}}. \end{array}$$

Specifically, *P* = [*P*_*x*_ *P*_*y*_ *P*_*z*_]^*T*^ denotes the three-dimensional linear momentum of the robot; *L* = [*L*_*x*_ *L*_*y*_ *L*_*z*_]^*T*^ denotes the three-dimensional angular momentum. *M*_jobarm_ and *H*_jobarm_ denote the inertia matrixes of the operating arm and ${\dot{\theta }}_{\text{jobarm}}$ denotes the velocity vector of the joints in the operating arm; *M*_aidarm_ and *H*_aidarm_ denote the inertia matrixes of the auxiliary arm while ${\dot{\theta }}_{\text{aidarm}}$ denotes the velocity vector of the joints in the auxiliary arm.

### 3.2. Full Balance and Six-Dimensional CMCR

The humanoid robot has six degrees of freedom; and, thus, the robot can achieve full balance only if it is well balanced and stable at every DOF. In other words, the robot is not expected to tilt or slide backward and forward or left and right, to jump vertically, or to rotate around the vertical axis.

The 6D CMCR is exactly corresponded to the 6 DOF balance maintenance of the humanoid robot. In the following part, the authors will illustrate the relation between the 6D CMCR and the 6 DOF balance maintenance, as well as how the full balance maintenance is achieved. It is straightforward that according to Newton's second law the momentum change rate equals the external force exerted by system. Since Newton's second law can also be applied to the generalized 6D form of force and moment, as discussed in this case, the momentum change rate of the humanoid robot has 6 dimensions of which the number equals the ground reactive force and moment acts on the robot feet. Specially, the robot will lose its balance and fails at its task, when the planned momentum change rate exceeds the maximum force and moment that can be exerted by the ground. The total momentum change rate of the robot can be formulated by time derivative of total momentum by using

(2) d[PL]dt=[dPxdtdPydtdPzdtdLxdtdLydtdLzdt]T=[F~xF~yF~zM~xM~yM~z]T.

Specifically, *t* denotes time; *dP*_*x*_/*dt*, *dP*_*y*_/*dt*, *dP*_*z*_/*dt*, *dL*_*x*_/*dt*, *dL*_*y*_/*dt*, and *dL*_*z*_/*dt* denote the momentum change rate of the six DOFs when the robot is moving. ${\tilde{F}}_{x}$, ${\tilde{F}}_{y}$, ${\tilde{F}}_{z}$, ${\tilde{M}}_{x}$, ${\tilde{M}}_{y}$, and ${\tilde{M}}_{z}$ represent the forces and moments the ground should provide to the robot feet. Obviously, the maximum force and moment the ground can provide to the robot by its feet is limited within a certain range, which is the CMCR range of the six DOFs. Note that the momentum change rate and the force and moment have the same dimension and are fundamentally identical. The constraint range in this paper is described by force and moment style while the momentum change rate style is also used. Therefore, the authors set out to regulate the force and moment within its constraints by calculating the momentum and controlling the momentum change rate, with the ultimate goal to achieve motion balance of the robot.

The authors will discuss and analyze the importance of momentum change rate at every dimension on balance, so as to find out the fundamental links between balance and momentum change rate.

(1) *dP*_*z*_/*dt* denotes the vertical component of the momentum change rate, which is equal to ${\tilde{F}}_{z}$ that the force the ground acts on the robot, representing the robot's vertical overweight and weight loss. The pressing force of the robot on the ground and the supporting force of the ground on the robot are a pair of interaction force. Specifically, the pressing force is *G* + *dP*_*z*_/*dt* with *G* being the weight of the robot. If *dP*_*z*_/*dt* > 0, the pressing force exceeds the robot's weight, and the robot presses the ground with its overweight. In this situation, the robot can provide greater static friction and can resist a greater tilting moment. Generally speaking, overweight is conducive to the robot to be steady; that is to say, the greater the *dP*_*z*_/*dt* the better. However, it has also been found that the greater overweight can exert negative effect on the robot, for it could enlarge the force on the robot structure and the moment on the joints. Thus, *dP*_*z*_/*dt* < 0.5*G* is defined.

If *dP*_*z*_/*dt* < 0, the pressing force is less than the weight of the robot; the robot loses its weight and attempts to escape from the ground. In this situation, the maximum static friction the ground can provide and the tilting moment it can resist will both decrease. Particularly, when *dP*_*z*_/*dt* = −*G*, the pressing force of the robot on the ground is zero. In this situation, the robot will leave the ground for weightlessness and thus is out of control. Since weightlessness is harmful to the balance of the robot, it needs to be under more rigorous control. *dP*_*z*_/*dt* > −0.3*G* is suggested.

Therefore, the vertical component of momentum change rate is constrained within the range as

(3) $$\begin{array}{c} -0.3G<\frac{dP_{z}}{dt}<0.5G. \end{array}$$

(2) *dL*_*x*_/*dt* and *dL*_*y*_/*dt* denote the tilting components of the momentum change rate in the directions of left and right and forward and backward, which are equal to moments ${\tilde{M}}_{x}$ and ${\tilde{M}}_{y}$ that the ground acts to the robot, representing to what degree the robot will tilt in the directions of left and right and forward and backward. When the tilting component of the momentum change rate is too large, the robot tends to tilt or even overturn. The degree of tilting is related to the tilting moment and the support margin in the specific direction. In practice, due to the flexibility of the robot structure and its joints, the tilting component of the momentum change rate will cause the robot swinging forward and backward or left and right. And the robot will actually tilt when it swings too violently.

The influence of tilting component of the momentum change rate on the robot's stability can be demonstrated by the disturbance on the ZMP point of the robot and defined as follows:

(4) $$\begin{array}{c} \Delta \text{ZM}{\text{P}}_{x}=\frac{-dL_{y}/dt}{G+dP_{z}/dt},\\ \Delta \text{ZM}{\text{P}}_{y}=\frac{dL_{x}/dt}{G+dP_{z}/dt}. \end{array}$$

Specifically, ΔZMP_*x*_ and ΔZMP_*y*_ are the shift of the ZMP point on the ground according to the tilting component of the momentum change rate. When the shift is so great that the ZMP point locates out of the support area on the ground, the robot is unstable, according to ZMP stability principle. In practice, the closer the ZMP point is to the edge of the support area, the less stable the robot will be. It is obvious that the dramatic changes of the tilting moment component will cause the ZMP point to deviate greatly from its original position. And at the same time, the robot will begin to shake, which will lead to actual tilting if the shake is too strong.

Assume that initially the ground projection of the robot's center of gravity is at the center of the support area and the length of the feet's support area in left-right plane and the forward-backward plane are defined as *A* and *B*; then

(5) $$\begin{array}{c} -\frac{B}{2}<\Delta \text{ZM}{\text{P}}_{x}<\frac{B}{2},\\ -\frac{A}{2}<\Delta \text{ZM}{\text{P}}_{y}<\frac{A}{2}. \end{array}$$

The constraint range of the tilting component of the momentum change rate is shown in (6) by taking (4) into (5):

(6) $$\begin{array}{c} -\frac{A}{2}\left(G+\frac{dP_{z}}{dt}\right)<\frac{dL_{x}}{dt}<\frac{A}{2}\left(G+\frac{dP_{z}}{dt}\right),\\ -\frac{B}{2}\left(G+\frac{dP_{z}}{dt}\right)<\frac{dL_{y}}{dt}<\frac{B}{2}\left(G+\frac{dP_{z}}{dt}\right). \end{array}$$

(3) *dP*_*x*_/*dt* and *dP*_*y*_/*dt* refer to the transitional sliding components of the momentum change rate to the forward and backward and the left and right, which are equal to ${\tilde{F}}_{x}$ and ${\tilde{F}}_{y}$ that the ground acts to the robot, representing to what degree the robot will transitionally slide on the ground. When the horizontal component of the momentum change rate is too great, the maximum static friction the ground can provide is not sufficient to support the horizontal momentum change. In this situation, the robot will slide transitionally.

The rigorous conditions on which the robot will not slide transitionally are defined as follows:

(7) $$\begin{array}{c} {\left(\frac{dP_{x}}{dt}\right)}^{2}+{\left(\frac{dP_{y}}{dt}\right)}^{2}<\mu^{2}{\left(G+\frac{dP_{z}}{dt}\right)}^{2}. \end{array}$$

Specifically, *μ* is the friction coefficient between the robot's feet and the ground. When the transitional sliding components of the momentum change rate do not satisfy this condition, the robot body will slide transitionally.

**Figure 2:**
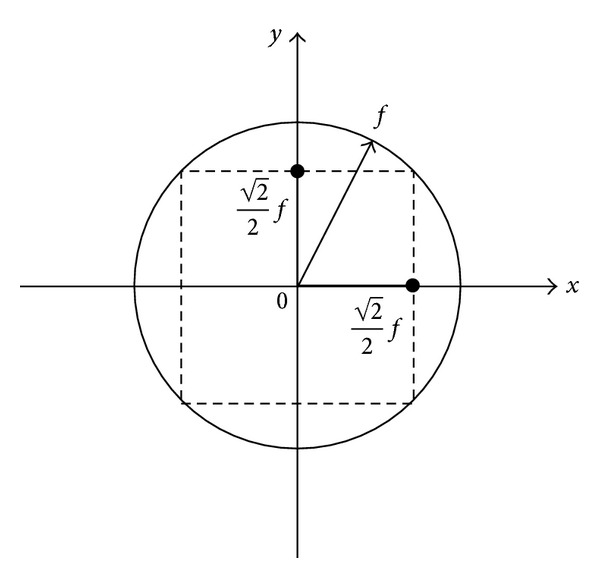
Linearization of transitional friction constraint.

According to the equation, (7) is a nonlinear inequality constraint and should be linearized for further discussion. According to common practice, the inequality circle constraint is replaced by the internal access square constraint, as shown in Figure 2.

Therefore, (7) can be replaced by the following linear inequality. It is obvious that the linearization is conservative, and the constraint range of transitional sliding of the momentum change rate is

(8) $$\begin{array}{c} -\frac{\sqrt{2}}{2}\mu \left(G+\frac{dP_{z}}{dt}\right)<\frac{dP_{x}}{dt}<\frac{\sqrt{2}}{2}\mu \left(G+\frac{dP_{z}}{dt}\right),\\ -\frac{\sqrt{2}}{2}\mu \left(G+\frac{dP_{z}}{dt}\right)<\frac{dP_{y}}{dt}<\frac{\sqrt{2}}{2}\mu \left(G+\frac{dP_{z}}{dt}\right). \end{array}$$

(4) *dL*_*z*_/*dt* is the rotating component of momentum change rate around the vertical axis, which is equal to moment ${\tilde{M}}_{z}$ that the ground acts to the robot, representing to what degree the robot will rotate around the vertical axis. When the rotating component of momentum change rate is too great, the maximum static friction the ground can provide is not enough to support the robot's momentum change in the rotation direction. In this situation, the robot will rotate around the vertical axis.

The accurate calculation of the maximum friction moment the ground can provide is fairly complex. As an engineering practice, the distributed pressing force the robot's two feet exert to the ground is assumed to be equal to the concentrated force exerted through the two feet's center point acts to the ground. Thus, the maximum friction force allowed is defined as

(9) $$\begin{array}{c} \frac{\mu C}{2}\left(G+\frac{dP_{z}}{dt}\right). \end{array}$$

Specifically, *C* is the distance between the centers of the two feet of the robot. Thus, the constraint range of the rotating component of the momentum change rate is

(10) $$\begin{array}{c} -\frac{\mu C}{2}\left(G+\frac{dP_{z}}{dt}\right)<\frac{dL_{z}}{dt}<\frac{\mu C}{2}\left(G+\frac{dP_{z}}{dt}\right). \end{array}$$

Based on the analyses above, the inequalities of the 6D momentum change rate to realize full balance maintenance of the humanoid robot are defined as

(11) $$\begin{array}{c} -\frac{\sqrt{2}}{2}\mu \left(G+\frac{dP_{z}}{dt}\right)<\frac{dP_{x}}{dt}<\frac{\sqrt{2}}{2}\mu \left(G+\frac{dP_{z}}{dt}\right)\\ -\frac{\sqrt{2}}{2}\mu \left(G+\frac{dP_{z}}{dt}\right)<\frac{dP_{y}}{dt}<\frac{\sqrt{2}}{2}\mu \left(G+\frac{dP_{z}}{dt}\right)\\ -0.3G<\frac{dP_{z}}{dt}<0.5G\\ -\frac{A}{2}\left(G+\frac{dP_{z}}{dt}\right)<\frac{dL_{x}}{dt}<\frac{A}{2}\left(G+\frac{dP_{z}}{dt}\right)\\ -\frac{B}{2}\left(G+\frac{dP_{z}}{dt}\right)<\frac{dL_{y}}{dt}<\frac{B}{2}\left(G+\frac{dP_{z}}{dt}\right)\\ -\frac{\mu C}{2}\left(G+\frac{dP_{z}}{dt}\right)<\frac{dL_{z}}{dt}<\frac{\mu C}{2}\left(G+\frac{dP_{z}}{dt}\right). \end{array}$$

It can be concluded from the inequalities of full balance conditions above that (1) either the tilting stableness represented by traditional ZMP or the jumping and sliding stableness is related to the momentum change rate of the corresponding dimension and (2) balance in any dimension is confined to a zone instead of a point. In other words, instability will occur when the momentum change rate is out of the zone. With smaller zone locating at the center of the support area, the bigger balance margin will bring stronger stability in practice. When the zone is narrowed down to a point, it falls into the traditional stability maintenance concept. For instance, in the direction of titling, when the zone is narrowed down to a point, it becomes the classical ZMP control. Most interestingly, though jumping in one direction, tiling in two directions, and sliding in three directions look like different phenomena, they can be defined in the same mathematical and physical constraint inequality.

By observing inequalities (11), we can find the overweight and weight loss of the robot coupled with the other five dimensions. To get rid of the coupling and to take into account the robot modeling errors and external disturbance, we will further tighten the constraint based on specific situations to get bigger balance margin. At first, weight loss coefficient *α* is added, and −*αG* ≤ *dP*_*z*_/*dt* ≤ 0.4*G* and 0 ≤ *α* < 0.3 are defined. *dP*_*z*_/*dt* = −*αG* is then taken into the five other constraints to get the constraint inequalities of the practical 6D constraints of momentum change rate:

(12) $$\begin{array}{c} -\frac{\sqrt{2}}{2}\mu \left(1-\alpha \right)G\leq \frac{dP_{x}}{dt}\leq \frac{\sqrt{2}}{2}\mu \left(1-\alpha \right)G\\ -\frac{\sqrt{2}}{2}\mu \left(1-\alpha \right)G\leq \frac{dP_{y}}{dt}\leq \frac{\sqrt{2}}{2}\mu \left(1-\alpha \right)G\\ -\alpha G\leq \frac{dP_{z}}{dt}\leq 0.4G\\ -\frac{A}{2}\left(1-\alpha \right)G\leq \frac{dL_{x}}{dt}\leq \frac{A}{2}\left(1-\alpha \right)G\\ -\frac{B}{2}\left(1-\alpha \right)G\leq \frac{dL_{y}}{dt}\leq \frac{B}{2}\left(1-\alpha \right)G\\ -\frac{\mu C}{2}\left(1-\alpha \right)G\leq \frac{dL_{z}}{dt}\leq \frac{\mu C}{2}\left(1-\alpha \right)G. \end{array}$$

Obviously, if inequalities in (12) are satisfied, inequalities in (11) must hold. And inequalities in (12) provide larger stability margins to get better balance performance.

The inequalities in (12) are transformed as follows for the convenience of further mathematical processing:

(13) $$\begin{array}{c} -\left[\begin{array}{c} F^{-}\\ M^{-} \end{array}\right]\leq \frac{d\left[\begin{array}{c} P\\ L \end{array}\right]}{dt}\leq \left[\begin{array}{c} F^{+}\\ M^{+} \end{array}\right], \end{array}$$

where

(14) $$\begin{array}{c} F^{-}={\left[\begin{array}{ccc} \frac{\sqrt{2}}{2}\mu \left(1-\alpha \right)G & \frac{\sqrt{2}}{2}\mu \left(1-\alpha \right)G & \alpha G \end{array}\right]}^{T},\\ F^{+}={\left[\begin{array}{ccc} \frac{\sqrt{2}}{2}\mu \left(1-\alpha \right)G & \frac{\sqrt{2}}{2}\mu \left(1-\alpha \right)G & 0.4G \end{array}\right]}^{T}, \end{array}$$

where *F* ^−^ and *F* ^+^ are 3D forces. Consider

(15) $$\begin{array}{c} M^{-}=M^{+}={\left[\begin{array}{ccc} \frac{A}{2}\left(1-\alpha \right)G & \frac{B}{2}\left(1-\alpha \right)G & \frac{\mu C}{2}\left(1-\alpha \right)G \end{array}\right]}^{T}, \end{array}$$

where *M* ^−^ and *M* ^+^ are 3D moments.

*F* ^+^ and *M* ^+^ denote the index of dynamic balance constraints of the robot's linear and angular momentum increase rates, which can also be called positive index of dynamic balance constraints. *F* ^−^ and *M* ^−^ are the index of dynamic balance constraints of the robot's linear and angular momentum decrease rates, which can be referred to as negative index of dynamic balance constraints.

### 3.3. Discretization and Optimization Solution of the CMCR

The formula of the CMCR needs to be discretized for convenient processing on the computer.

Firstly, the derivative of momentum in inequalities (13) is discretized with a sample time of *T*. Consider

(16) $$\begin{array}{c} -\left[\begin{array}{c} F^{-}\\ M^{-} \end{array}\right]\leq \frac{{\left[\begin{array}{c} P\\ L \end{array}\right]}_{i}-{\left[\begin{array}{c} P\\ L \end{array}\right]}_{i-1}}{T}\leq \left[\begin{array}{c} F^{+}\\ M^{+} \end{array}\right]. \end{array}$$

Then, the robot's total momentum at the *i*th cycle is taken into the two arms' momentum formula. Consider

(17) −T∗[F−M−]≤([MjobarmHjobarm]θ˙jobarm+[MaidarmHaidarm]θ˙aidarm)i−[PL]i−1≤T∗[F+M+].

Lastly, the auxiliary arm's momentum formula at the *i*th cycle is kept in the middle of the inequalities. Since the inertia matrix of the auxiliary arm is not square matrix, let alone nonsingular matrix, and thus it does not need to be simplified. Therefore

(18) [PL]i−1−([MjobarmHjobarm]θ˙jobarm)i−T∗[F−M−] ≤([MaidarmHaidarm]θ˙aidarm)i ≤[PL]i−1−([MjobarmHjobarm]θ˙jobarm)i+T∗[F+M+].

The angular velocity vector of the joints in the auxiliary arm that fits with the constraint inequality (18) can realize all-dimensional CMCR at the six degrees of freedom; in other words, the robot can achieve overall balance and stability. However, multiple solutions of the angular velocity vector of the joints in the auxiliary arm that fits with the constraint inequalities exist. Thus, it is necessary to set the angular velocity of the joints in the auxiliary arm as the optimal goal by means of convex quadratic programming, in order to get the only optimal vector. The mathematic description of the inequalities constrained by the convex quadratic programming is defined as

(19) min⁡x12xTQx,subject  to b1≤A0x≤b2.

Specifically, *Q* is the optimal weight matrix of the angular velocity of the joints in the auxiliary arm; usually unit diagonal matrix is competent. Consider

(20) $$\begin{array}{c} x={\left({\dot{\theta }}_{\text{aidarm}}\right)}_{i},\\ A_{0}={\left(\left[\begin{array}{c} M_{\text{aidarm}}\\ H_{\text{aidarm}} \end{array}\right]\right)}_{i},\\ b_{1}={\left[\begin{array}{c} P\\ L \end{array}\right]}_{i-1}-{\left(\left[\begin{array}{c} M_{\text{jobarm}}\\ H_{\text{jobarm}} \end{array}\right]{\dot{\theta }}_{\text{jobarm}}\right)}_{i}-T*\left[\begin{array}{c} F^{-}\\ M^{-} \end{array}\right],\\ b_{2}={\left[\begin{array}{c} P\\ L \end{array}\right]}_{i-1}-{\left(\left[\begin{array}{c} M_{\text{jobarm}}\\ H_{\text{jobarm}} \end{array}\right]{\dot{\theta }}_{\text{jobarm}}\right)}_{i}+T*\left[\begin{array}{c} F^{+}\\ M^{+} \end{array}\right]. \end{array}$$

### 3.4. Motion Constraint of the Auxiliary Arm Joints

In practice, it is necessary to take into account the output angular velocity and acceleration of the motor and reducer of the joints in the auxiliary arm. If not, the motor will overspeed and be overheated for the large current, and the reducer will overload. Since the constraint conditions of the defined convex quadratic programming are open, more reasonable constraints can be added. In the following part, the motion constraint of the joints in the auxiliary arm will be formulated.

Assume that the angular velocity constraint of the joints in the auxiliary arm is ${\dot{\theta }}_{\max}$ and the velocity constraint on the angular acceleration is *a*, and then the velocity constraint on the joints is defined as

(21) $$\begin{array}{c} -{\dot{\theta }}_{\max}\leq x\leq {\dot{\theta }}_{\max}. \end{array}$$

The constraint on the angular acceleration of the joints will be

(22) $$\begin{array}{c} {\left({\dot{\theta }}_{\text{aidarm}}\right)}_{i-1}-a*T\leq x\leq {\left({\dot{\theta }}_{\text{aidarm}}\right)}_{i-1}+a*T. \end{array}$$

Above all, the motion constraint of the joints in the auxiliary arm is

(23) $$\begin{array}{c} c_{1}\leq x\leq c_{2}. \end{array}$$

Specifically, $c_{1}=\max(-{\dot{\theta }}_{\max},{\left({\dot{\theta }}_{\text{aidarm}}\right)}_{i-1}-a*T)$ means that the maximum in the lower limits of the angular velocity constraints of every joint while $c_{2}=\min({\dot{\theta }}_{\max},{\left({\dot{\theta }}_{\text{aidarm}}\right)}_{i-1}+a*T)$ refers to the minimum in the upper limits of the angular velocity constraints of every joint.

### 3.5. Algorithm

The convex quadratic programming falls into the category of classical mathematic programming with mature algorithms. Since the problem scale is small, the following steps have been taken to improve the speed of algorithm.

1. The kinematic chain of calculation is shortened. The desired motion and balance maintenance of the robot is and can only be performed by the two arms with other joints in the body remaining fixed. Thus, only the motion of the two arms needs to be calculated.
2. Array structure, instead of tree structure, is used in the algorithm data structure. The traverse calculation can be completed by one simple linear loop.
3. Recursive algorithm is replaced by nonrecursive algorithm, in order to save the calculation overhead and increase the speed.

Based on the steps above, the effectiveness and real-time performance of the algorithm have been tested on a computer platform with the configuration of Intel Core 2 Duo 2.93 GHz CPU, 2 GB RAM, Windows Xp. The computational time for each sample cycle on Matlab R2008b is 5.6 ms on average and 8.6 ms at most. Thus, the computation speed of the algorithm basically meets the requirement of real-time applications.

## 4. Simulation Results

**Figure 3:**
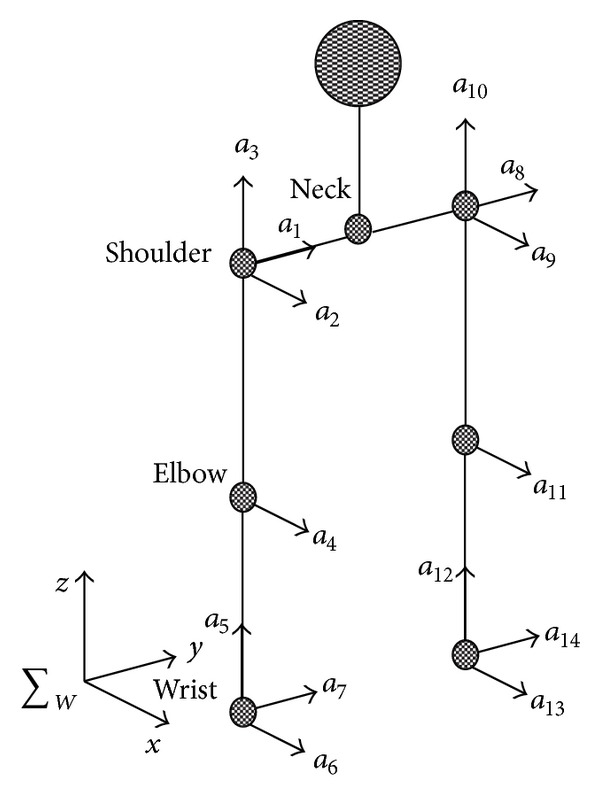
Model structures of the robot arms.

We have simulated the high-speed motion of the humanoid robot's right arm for further experiments and analyses. The robot model we used in the experiment is shown in Figure 1. The robot weighs 55 kg, with a height of 165 cm and 30 degrees of freedom. Specifically, the robot has 7 degrees of freedom in its two arms, respectively, 3 in its shoulders, 1 in its elbow, and 3 in its wrist.  Figure 3 shows the structure of the two arms, with detailed parameters in Table 1 and the motion parameters of each joint of the arms in Table 2.

The constraint parameters are set as follows. Based on previous experience, the weight loss coefficient is *α* = 0.15 and then the dynamic balance constraint index has been calculated as follows:

(24) F−=[979780]T NM−=[1035420]T NmF+=[9797215]T NM+=[1035420]T Nm.

According to test results, though the dynamic balance constraint index can guarantee basic balance and stableness, the constraint is still too loose. To achieve better balance and stability, the constraint is further tightened as

(25) $$\begin{array}{c} \left[\begin{array}{c} F^{-}\\ M^{-} \end{array}\right]={\left[\begin{array}{cccccc} 97 & 97 & 80 & 80 & 40 & 20 \end{array}\right]}^{T},\\ \left[\begin{array}{c} F^{+}\\ M^{+} \end{array}\right]={\left[\begin{array}{cccccc} 97 & 97 & 188 & 80 & 40 & 20 \end{array}\right]}^{T}. \end{array}$$

For uniformity and writing convenience, the force and moment are unified into 6D column vector with their units removed.

The angular velocity constraint and angular acceleration constraint of the joints in the auxiliary arm are the maximum angular velocity and maximum angular acceleration in Table 2.

**Figure 4:**
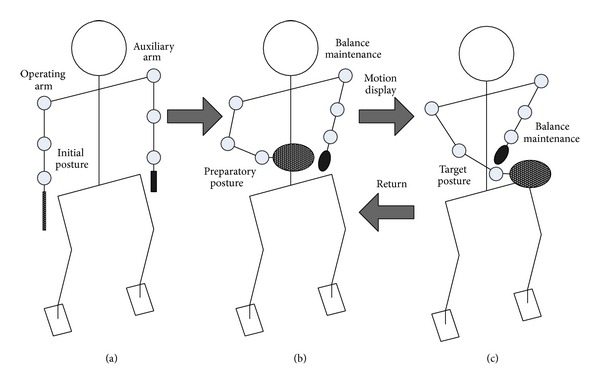
Operation and balance maintenance processing of the humanoid robot.

In the simulation experiment, the task of the humanoid robot's operating arm (the right arm) performs the motion as follows: at first, the operating arm returns to the initial posture shown in Figure 4(a); then, the operating arm moves to the preparatory posture in Figure 4(b) with the time duration of 0.3 s; later, the operating arm waits for the task for 0.3 s in this case. Generally, the task means that the operating arm moves from the preparatory posture to the designated target posture within specified time duration at the designated target speed. In this paper, the set time is 0.3 s and the designated target posture is as shown in Figure 4(c). Moreover, the designated target speed includes linear velocity and angular velocity; specifically, the linear velocity is [1.76 0 0.38]^*T*^ m/s, the angular velocity [0 0 0]^*T*^ rad/s, the synthesized linear velocity 1.8 m/s, and synthesized angular velocity 0 rad/s. After the designated task gets finished, the operating arm returns to its preparatory posture from the target posture, waiting for the next task.

Relatively great continuous inertia impact is exerted to the robot body when the robot's arm accelerated from static to the synthesized linear velocity up to 1.8 m/s in a short time of 0.3 s. However, the greatest impact does not occur when the maximum synthesized linear velocity is reached; instead, it happens after the arm reaches the maximum synthesized linear velocity and when it is ready to withdraw its arm. At this moment, the main joints of the operating arm switch the maximum angular acceleration to the opposite maximum angular acceleration in an instant. Large jerk value is required bringing instantaneous impact to the humanoid robot body, and therefore the balance maintenance of the robot will play a vital role. As a result, it can be concluded that balance maintenance of the auxiliary arm is needed only when the operating arm performs and withdraws. From initial posture to preparatory posture, the time interval is long enough and the motion is mild, so this time interval is not the focus of this paper. Therefore, at this interval, the auxiliary arm is not used for balance maintenance but for moving to an advantageous initial posture.

From Figures 6 and 7, the angular accelerations and angular curves of the 7 joints of the robot's right arm show the motion of the operating arm in the whole process of the task. To put it more specifically, in Figure 6, the operating arm reaches the preparatory posture, the planned target posture, and the withdrawal preparatory posture at times 0 s, 0.3 s, and 0.8 s, respectively. At the same time, balance maintenance of the robot's auxiliary arm is executed simultaneously to guarantee the balance of the whole body.

**Figure 5:**
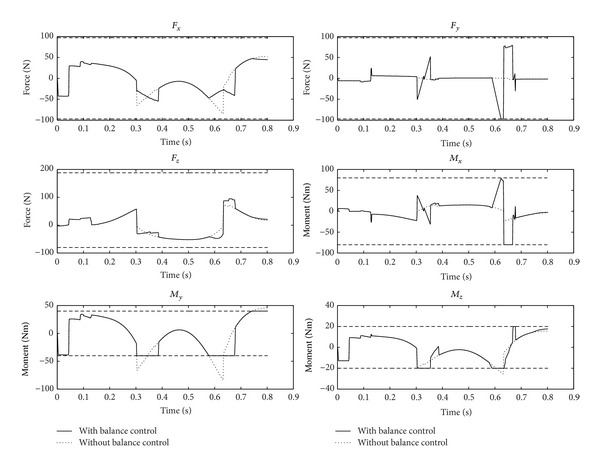
Momentum change rate curve of the robot.

Figure 5 demonstrates that the 6D constraints of momentum change rate of the robot is up to the expectation, for the fact that the 6D momentum change rate is constrained within the planned range of balance. Here we present several detailed problems: (1) *F*_*x*_ and *F*_*z*_ are not greatly influenced by the existence of balance maintenance, and these two dimensions are still within the stability range; (2) *M*_*y*_ and *M*_*z*_, which are the two dimensions to which the constraint of momentum change rate exerts active and effective effect, remain within the stability range after the balance maintenance has been executed. (3) Unfortunately, *F*_*y*_ and *M*_*x*_ increase so greatly after balance maintenance is executed that their value almost reaches the range of stability constraints, but they are still controlled within the constraint range. This shows the concept proposed in this paper that balance maintenance is a matter of range and only needs to be constrained only when the robot attempts to exceed the range of stability. In addition, it is clear that the humanoid robot is a severely nonlinear and strong coupling system. Under the worst circumstances, changes in some parts will lead other parts to change dramatically or even to worsen. It is necessary to take into consideration this feature when designing the balance maintenance strategy.

**Figure 6:**
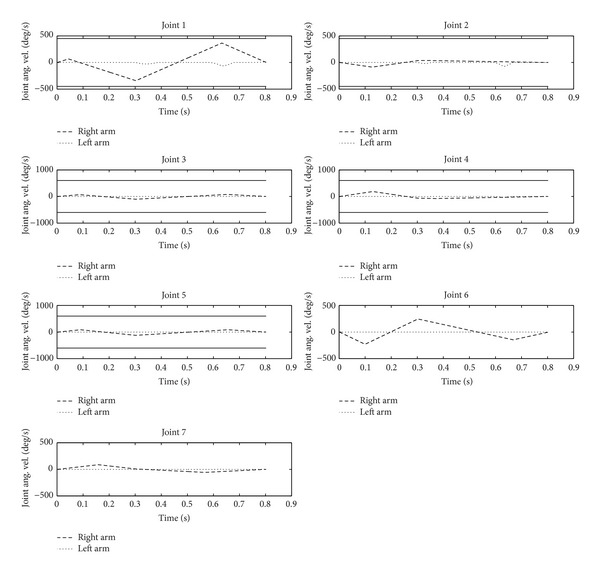
Angular velocity curve of the robot's arms.

Figure 6 shows the curve of the angular velocity of the operating arm's joints and the auxiliary arm's joints generated by the full balance maintenance. It can be seen that the auxiliary arm moves only near at 0.3 s and 0.6 s, which are the exact moments when the angular velocity of the operating arm's joints reaches the maximum and the angular acceleration switches to the opposite direction. This fully shows that the impact generated by the operating arm is not only related to the angular velocity of the joints but, more importantly, also greatly linked to the angular acceleration. Thus, it is more necessary to focus on the angular acceleration, instead of the angular velocity of the joints in the auxiliary arm, so as to resist the impact of the operating arm. As a result, the angular velocity curve of the auxiliary arm's joints is steeper.

**Figure 7:**
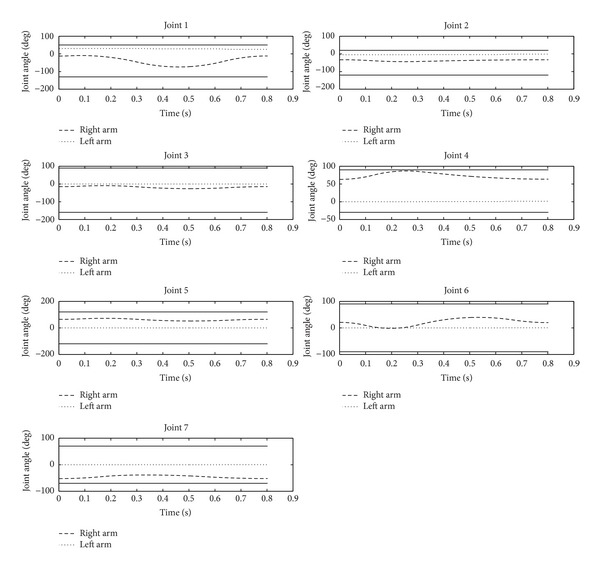
Joint angular curve of the robot's arms.

Figure 7 shows the angular curve of the operating arm's joint as well as the angular curve of the auxiliary arm's joint generated by the balance maintenance. The angular curve of the joint is the integration of its angular velocity curve, so it is relatively smooth. Note that the auxiliary arm helps balance maintenance within a short period of time, and thus the angular change of the joints in the auxiliary arm is minor with great angular margin.

**Figure 8:**
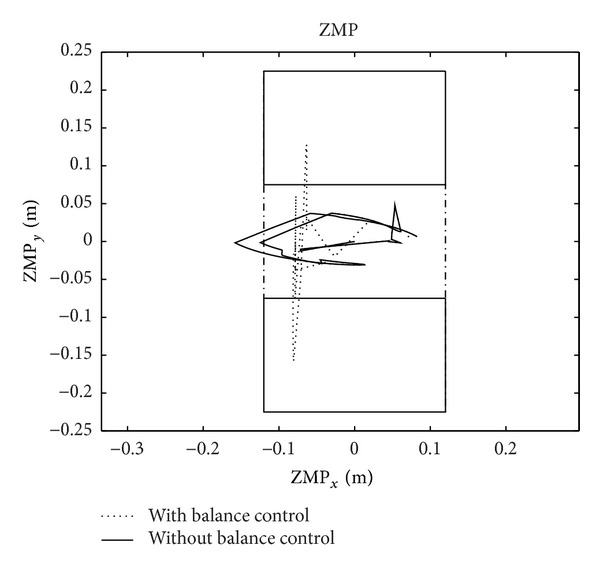
ZMP curve of the robot.

Figure 8 is the comparison of the ZMP curves under the robot's feet when the robot is performing the same task as mentioned and after full balance maintenance is exerted. The solid line shows the ZMP curve without balance maintenance of the auxiliary arm. From this curve, ZMP under the robot's feet obviously exceeds the support area. According to ZMP theory [14], the operation of the robot is unstable; in other words, the robot will fall and fail at the motion when the ZMP exceeds. The dotted line shows the ZMP curve under the robot's feet when the operation and auxiliary arms work together. With balance maintenance, ZMP is fully controlled within the support area, with a certain distance kept from the edge of the support area. According to ZMP theory [14], the robot's operation is stable, with a certain amount of stability margin.

The ZMP curve after balance maintenance has been executed can be observed from the other aspect. In Figure 5, *F*_*z*_, *M*_*x*_, and *M*_*y*_ are successfully constrained within the range of stability; in other words, they respectively meet the third, fourth, and fifth conditions in constraint inequalities (12), which can guarantee the ZMP stability of the robot. Therefore, the traditional ZMP stability is just a subset of the full balance maintenance method put forward in this paper. It is evident that the full balance maintenance proposed in this paper includes ZPM stability, as well as the balance on the six degrees of freedom which can make sure of the comprehensive stability of the robot.

We have selected ten groups of typical motion programming data of the operating arm. The expected effect has been achieved in these tests by means of the full balance maintenance method, employed with the constraints of momentum change rate and joint motion of the auxiliary arm. The test involves 12 dynamic balance constraint indexes, angular acceleration constraints, and angular velocity constraints of the seven joints in the robot's auxiliary arm, as well as the optimal diagonal weight matrix parameters. As a result, we can conclude the following.

Firstly, balance maintenance during high-speed motion of the robot under the constraint of the momentum change rate is feasible; and the framework of full balance maintenance is open and extensive. For instance, angular velocity and angular acceleration constraints on the joints of the auxiliary arm have been added in this paper. In other words, other reasonable requirements can be added to the solution, as long as they can be written in the form of constraint inequalities. For example, collision and interference constraints of the auxiliary arm can be added if necessary.

Secondly, the multiple parameters in the convex quadratic programming constrained by the inequalities on the one hand stand in the way of full balance maintenance; but, on the other hand, they bring great flexibility to the realization of the convex quadratic programming. For instance, these parameters can be organically linked by means of neural network or even learned by iterative evolution algorithm, since these parameters can be adjusted online at real time.

Lastly, full balance maintenance is a concept and constraint of momentum change rate is an idea. The convex quadratic programming under the constraint of the inequalities is one of the mathematic methods under this concept and this idea. It is possible that a better and more advanced mathematical tool can be sought to achieve better balance between balance maintenance and the motion constraint of the auxiliary arm's joints.

All in all, the concept of full balance and the idea of constraint of momentum change rate is a new solution to achieve dynamic balance at the six degrees of freedom when the humanoid robot is performing high-speed motion. The solution has been specifically illustrated and briefly experimented in the paper and shows a good generality. In addition, this solution is highly open, extensive, and flexible in the parameter adjustments and realization methods. Thus, the authors have created a new framework for the balance and stability of the robot, with great value for future studies.

To test the balance performance when the robot operating arm's end is at a higher speed, we gradually increase the linear speed at the end of the operating arm within the range of the robot's actual capabilities. It has been found that, when the linear speed is as high as 3.2 m/s, the robot can still remain balanced. Moreover, the bottleneck that occurs first is the overrunning of the angular velocity of the joints to their limits in the operating arm, rather than those in the auxiliary arm. As a matter of fact, the auxiliary arm modifies its angular acceleration to adjust robot momentum change rate for balance only when the momentum change rate tries to exceed the dynamic balance constraint index, and this process will not last long. Thus, the angular velocity of the auxiliary arm basically will not overrun. However, the angular acceleration of the joints in the auxiliary arm should not be too large or last with a high speed for too long. The robot's arm end can perform with balance at a higher speed, if the actual performance capacities of the robot arm are further improved.

## 5. Conclusion

This paper puts forward the concept of full balance maintenance, as well as the idea of constraint of momentum change rate for the first time, and the concept and idea have been employed to achieve balance maintenance of the high-speed motion of the humanoid robot. Firstly, the total momentum formula of the robot's two arms is provided. Then momentum change rate is defined by the time derivative of the total momentum. In addition, the authors illustrate the concept of full balance, so as to find out the physical meaning of the constraint of the momentum change rate at the six dimensions, as well as the relation between full balance and 6D CMCR. Lastly, for the convenience of computer processing, CMCR is discretized and the convex quadratic programming is employed to solve motion constraint of the joints in the auxiliary arm. Besides, the actual algorithm is optimized to get real-time performance. The simulation results show the validity to achieve balance maintenance at the six degrees of freedom of the robot body by means of 6D CMCR. The resulted balance motion of the auxiliary arm has large angular margin, maintains angular velocity and angular acceleration within the range, and archives a comprehensive dynamic balance performance and good ZMP stability margin. The actual tests have a good real-time performance. For future research, the real-time full balance maintenance utilizing the motion of the humanoid's waist and legs' joints will be studied.

## Figures and Tables

**Table 1:** Detailed parameters of the arms.

Arms	Length (m)	Mass (kg)	Equivalent cross-section size (m ∗ m)
Upper arm	0.25	3.5	0.1∗0.1
Forearm	0.25	2.5	0.08∗0.08
Hand	0.20	0.5	0.05∗0.05

**Table 2:** Motion parameters of the arm joints.

	Motion range of the joint angle (deg)		Maximum angular velocity (deg/s)	Maximum angular acceleration (deg/s^2^)
	Minimum	Maximum		
Pitch of shoulder	−120	40	430	5730
Roll of shoulder	−130	10	430	5730
Yaw of shoulder	−170	90	573	5730
Roll of elbow	−20	110	573	5730
Yaw of wrist	−130	130	573	2865
Roll of wrist	−90	90	487	2865
Pitch of wrist	−60	60	487	2865
